# Transforming Dental Caries Diagnosis Through Artificial Intelligence-Based Techniques

**DOI:** 10.7759/cureus.41694

**Published:** 2023-07-11

**Authors:** Sukumaran Anil, Priyanka Porwal, Amit Porwal

**Affiliations:** 1 Dentistry, Hamad Medical Corporation, Doha, QAT; 2 Dentistry, Pushpagiri Institute of Medical Sciences and Research Centre, Tiruvalla, IND; 3 Prosthetic Dental Sciences, College of Dentistry, Jazan University, Jazan, SAU

**Keywords:** clinical applications, performance evaluation, data acquisition, convolutional neural networks, image analysis, machine learning, artificial intelligence, dental radiographs, dental caries diagnosis

## Abstract

Diagnosing dental caries plays a pivotal role in preventing and treating tooth decay. However, traditional methods of diagnosing caries often fall short in accuracy and efficiency. Despite the endorsement of radiography as a diagnostic tool, the identification of dental caries through radiographic images can be influenced by individual interpretation. Incorporating artificial intelligence (AI) into diagnosing dental caries holds significant promise, potentially enhancing the precision and efficiency of diagnoses. This review introduces the fundamental concepts of AI, including machine learning and deep learning algorithms, and emphasizes their relevance and potential contributions to the diagnosis of dental caries. It further explains the process of gathering and pre-processing radiography data for AI examination. Additionally, AI techniques for dental caries diagnosis are explored, focusing on image processing, analysis, and classification models for predicting caries risk and severity. Deep learning applications in dental caries diagnosis using convolutional neural networks are presented. Furthermore, the integration of AI systems into dental practice is discussed, including the challenges and considerations for implementation as well as ethical and legal aspects. The breadth of AI technologies and their prospective utility in clinical scenarios for diagnosing dental caries from dental radiographs is presented. This review outlines the advancements of AI and its potential in revolutionizing dental caries diagnosis, encouraging further research and development in this rapidly evolving field.

## Introduction and background

Dental caries is a widespread disease that affects an estimated 3.5 billion individuals worldwide, making it one of the most common human afflictions. It impacts between 60% and 90% of children and the majority of adults globally, thereby causing significant financial strain and impacting the quality of life [1,2]. Dental caries is a complex disease caused by plaque, leading to dental hard tissue deterioration. It is a dynamic process in which salivary proteins attach to the tooth surface, setting the stage for initial plaque formation by providing a substrate for bacterial adhesion. This adhesion results in demineralization due to acid production by bacteria in dental plaque biofilms, which leads to caries [3]. *Streptococcus mutans*, a chief bacterium involved in tooth decay, can integrate into biofilm substrates, forming a highly acidic microenvironment with a pH below 5.0. This acidity dissolves hard tooth apatite, setting the stage for tooth decay [4].

Detecting dental caries at the initial stages can avert the need for invasive procedures and significantly reduce healthcare expenses [5]. However, identifying initial proximal caries solely through clinical examinations is challenging, making bitewing radiography a valuable tool, as it is considered the gold standard for diagnosing demineralized proximal caries. Visual inspection and bitewing radiographs are typically used for routine detection of proximal caries. Other detection methods include fiberoptic transillumination, fluorescence-based techniques, and cone-beam computed tomography (CBCT) [6]. Despite these alternatives, each method has shortcomings, such as limitations in detecting posterior initial proximal caries and additional costs for the required devices. Thus, bitewing radiography continues to be the most reliable and commonly applied method in clinical settings [7].

Among these challenges, a new hope has emerged in the form of artificial intelligence (AI), which is poised to revolutionize many aspects of healthcare, including dental caries diagnosis [8-10]. AI, a branch of computer science that simulates human intelligence processes by machines, especially computer systems, is changing the game by enabling more accurate, efficient, and personalized care. AI in healthcare has seen a surge of interest in recent years. It includes various applications such as predicting disease onset, enabling precise surgery, personalizing treatment plans, managing patient care, and augmenting nursing capabilities [11]. In dentistry, AI capabilities can address the challenges associated with the traditional methods of dental caries diagnosis. Machine learning algorithms can analyze extensive amounts of data, and with an increasing number of dental images available, the application of AI in dental caries diagnosis appears promising [12]. Machine learning algorithms can interpret dental images such as radiographs, photographs, and three-dimensional scans more accurately and consistently than human eyes, thus reducing the subjective variability associated with traditional diagnostic methods [13]. Furthermore, the ability of AI to identify patterns in large datasets can lead to the early detection of caries, even before visible signs become clear to the human eye. The potential of AI in dental caries diagnosis is not limited to merely identifying the disease; it can also contribute to predicting disease progression and treatment outcomes, providing a more comprehensive approach to dental care [14]. This involves a shift from a reactive model of care to a preventive and personalized approach in alignment with modern healthcare philosophy.

## Review

Artificial intelligence algorithms used for dental caries detection

AI, particularly machine learning and deep learning algorithms, has shown promising results in detecting and diagnosing dental caries. These algorithms use vast amounts of dental image data to learn the patterns and characteristics associated with caries, enabling the viewing of the condition in new, unseen images accurately [15]. Predictive modeling leverages data and statistical algorithms to forecast outcomes with optimal accuracy. The incorporation of AI, especially machine learning algorithms, has greatly enhanced the strength and precision of predictive modeling. A systematic review deduced that deep learning models, employing a wide array of architectures, can prove instrumental in aiding dentists in the detection of caries lesions [16].

Convolutional Neural Networks in Image Analysis and Caries Detection

Convolutional neural networks (CNNs) are a category of AI designed to automatically and adaptively learn spatial hierarchies of features directly from images, making them exceptionally well suited for tasks such as image classification, object detection, and semantic segmentation [17]. CNNs, with their ability to learn from a large amount of annotated data, offer a solution to these challenges by bringing more precision and consistency to the task [18]. CNNs operate by learning an internal representation of an image and focusing on local spatial hierarchies. This ability to recognize localized features within an image sets CNNs apart from other machine learning techniques. Once a CNN model is trained on annotated dental images, it can accurately detect and classify dental caries in new images [19]. By identifying intricate patterns that the human eye can overlook, CNNs can highlight potential areas of concern, enhancing diagnostic accuracy. CNN trained with the Edge Extraction strategy performed excellently in detecting proximal caries on periapical radiographs. Different training strategies, such as image preprocessing, can be considered to improve the accuracy of the CNN model, especially when a small dataset is used [20]. A comprehensive systematic review has indicated that deep learning CNNs exhibit substantial precision in identifying and diagnosing dental caries [21].

Moreover, CNNs can process several dental images in a short time, increasing the efficiency of dental caries detection. This ability is particularly beneficial in high-volume dental clinics where the timely interpretation of images can directly impact patient turnover and overall clinic productivity [22]. However, while using CNNs in dental caries detection is promising, it also presents several challenges. These include issues related to data privacy and security, the need for large, well-annotated datasets for training the CNNs, and the requirement for rigorous validation of the models. CNNs offer significant potential in revolutionizing dental caries detection by enhancing diagnostic accuracy, improving efficiency, and offering predictive insights. As the field continues to evolve and mature, with appropriate attention to regulatory, ethical, and data considerations, CNNs are poised to become a staple in modern dental imaging analysis and caries detection [23].

CariesNet is a proposed deep learning model designed to enhance the diagnostic process of dental caries. Built upon a CNN architecture, CariesNet utilizes AI and machine learning to analyze dental radiographs and accurately identify the presence of dental caries. This AI-driven approach has shown significant potential in improving the accuracy and efficiency of dental caries diagnosis compared to traditional manual inspections [24]. It performs a pixel-level classification, distinguishing carious lesions from healthy tissue, which enables dentists to determine the precise location and extent of decay. The model is trained with a vast dataset of annotated dental X-rays, which include a wide range of patient ages, types of teeth, and stages of caries. Through rigorous training, the model learns to differentiate dental caries from healthy dental tissues and other dental anomalies.

Support Vector Machines for Enhanced Dental Caries Diagnosis

Support vector machines (SVMs) are powerful machine learning algorithms primarily used for classification and regression tasks. SVMs have been extensively applied in various fields of biomedical sciences, including dentistry [25]. The strength of SVMs lies in their ability to handle high-dimensional data, their robustness against overfitting, and their ability to manage nonlinear classification problems through kernel functions. In the context of caries diagnosis, SVMs can be highly effective. The diagnosis of dental caries typically involves analyzing various data, including dental images, patient history, and potentially genomic or other biological data. SVMs are well suited to this task because they can handle multiple types of data simultaneously and can recognize complex patterns within the data that might not be easily discernible to a human observer [26]. Once the SVM model is trained, it can diagnose new, unseen patients. Given the dental record of a new patient, the SVM model predicts whether the patient shows signs of dental caries based on what it has learned from the training data [27].

Moreover, SVMs offer robustness against overfitting, a common problem in machine learning where a model learns the training data too well and performs poorly on new data. However, while SVMs offer promising potential in dental caries diagnosis, their application is not without challenges. One challenge is the need for a large representative training dataset. Another challenge relates to the interpretability of SVMs. SVM models, particularly those employing nonlinear kernel functions, can be challenging to interpret, which may pose problems in a clinical context where interpretability can be crucial for treatment decisions. SVMs can play a significant role in diagnosing dental caries, given their ability to handle high-dimensional data, robustness against overfitting, and capacity to manage complex, nonlinear classification problems [28].

Random Forest Algorithm for Improved Dental Caries Detection

Random forests is a machine learning algorithm widely used for classification and regression tasks owing to its simplicity, versatility, and robust performance. This ensemble learning method operates by constructing numerous decision trees at training time and outputting the class, that is, the mode of the categories (classification) or mean prediction (regression) of the individual trees [29]. In the context of dental caries detection, random forests can be a valuable tool. The capacity of random forests to manage high-dimensional data and effectively handle missing or unbalanced data makes them a potent asset in such analyses. Random forests offer a unique advantage over other machine learning techniques. While many algorithms struggle with overfitting, i.e., performing well on training data but poorly on unseen data, random forests demonstrate inherent resistance to overfitting. This characteristic is precious in medical applications where the accurate prediction of new data is crucial [30]. Unlike some machine learning models that act as a “black box,” random forests provide insights into which features are essential in predicting dental caries. This interpretability can guide dentists in understanding risk factors and tailoring patient preventive measures [31,32].

Deep Learning for Dental Caries Based on the ICDAS™ Radiographic Scoring System

The International Caries Detection and Assessment System (ICDAS™) is a universally recognized system for the detection and classification of dental caries. It provides a standardized approach to caries detection, making it easier for dental professionals to communicate and compare findings [33]. The ICDAS™ provides a scoring system for dental caries that ranges from 0 to 6, reflecting the extent of decay, with 0 representing healthy tooth surfaces and 6 denoting extensive, distinct cavities. The application of deep learning techniques for dental caries classification, built on the foundation of the ICDAS™ scoring system, holds substantial promise. Such an approach can significantly enhance both the precision and the speed of dental caries diagnosis [34].

Image processing and analysis techniques for identifying caries lesions

Identifying caries lesions in dental radiographs involves several key steps, ranging from the initial image acquisition to advanced image processing and analysis [35].

Image Acquisition

This is the initial phase where bitewing radiography, orthopantomogram, or other imaging techniques are used to capture dental images. It is crucial to ensure high-quality images to improve the accuracy of subsequent analysis.

Pre-processing

This step aims to improve the quality of the image and make it more suitable for subsequent analysis. Pre-processing techniques may include those listed below.

Noise reduction: Various filters, such as Gaussian smoothing or median filters, can reduce noise.

Contrast enhancement: Techniques such as histogram equalization or adaptive histogram equalization can improve the image contrast, making the caries lesions more visible.

Segmentation

This involves dividing the image into different regions or segments. Thresholding is a common technique used in dental imaging, where pixels are classified as either “tooth” or “not tooth” based on their intensity.

Feature Extraction

Features that can help identify caries are extracted from the pre-processed images.

Intensity-based features: These are based on the pixel values in the caries region, which tend to be lower (indicating a darker region) than healthy tooth tissue.

Texture-based features: These are based on patterns and variations in pixel intensities within the caries region, which may have a distinct texture compared to healthy tissue.

Shape-based features: These could involve the shape and size of suspected caries lesions.

Classification

After feature extraction, a classifier is used to identify whether the extracted features represent caries. Various machine learning and AI algorithms can be used, such as SVMs, random forests, or deep learning models such as CNNs [27]. The classifier is usually trained on labeled data (i.e., images where the presence or absence of caries has been confirmed).

Post-processing

This final step involves refining the classification results to improve accuracy. This can include removing small isolated regions identified as caries that may be likely noise or artifacts.

Validation

The performance of the entire image processing and analysis pipeline should be validated using an independent test set. Performance measures such as sensitivity, specificity, precision, recall, and F1 score can be calculated to evaluate the effectiveness of image-processing techniques in identifying caries lesions.

**Figure 1:**
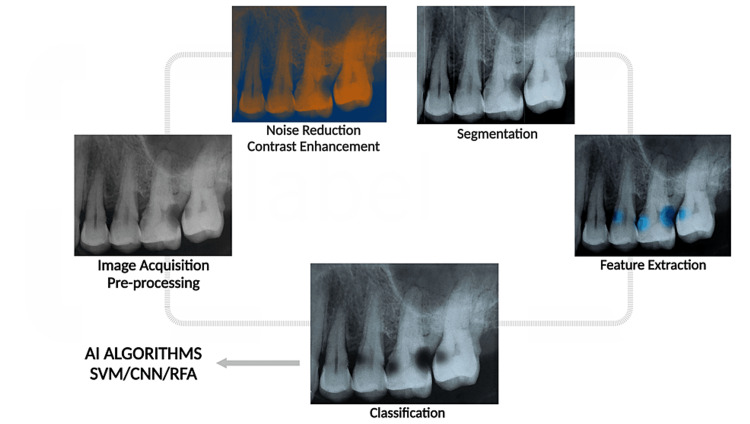
The process of analyzing dental radiographs and identifying dental caries through artificial intelligence. SVM = support vector machine; CNN = convolutional neural network; RFA = random forest algorithm

The process of analyzing dental radiographs and identifying dental caries is illustrated in Figure 1.

Ethical and legal challenges in artificial intelligence-based diagnosis of dental caries

As AI continues to permeate various healthcare domains, its role in dentistry, particularly in diagnosing dental caries, is increasingly noteworthy. While AI-based diagnosis promises to revolutionize dental care, it presents a unique ethical and legal challenge [36].

Data Privacy and Security

AI models require vast data for training, validation, and testing. In dentistry, these data include highly sensitive patient information such as dental images, medical histories, and demographic details. Ensuring the privacy and security of this data is paramount, requiring robust data protection measures and adherence to regulations such as the General Data Protection Regulation in Europe or the Health Insurance Portability and Accountability Act in the United States [37].

Informed Consent

Using patient data for training AI systems necessitates informed consent. Patients must know how their data will be used and the potential risks [38]. The complexity of AI may pose challenges to achieving truly informed consent, requiring efforts to educate patients about the technology.

Accountability and Liability

One of the critical legal considerations in AI-based dental caries diagnosis is determining liability in case of misdiagnosis or treatment failure. As AI systems become more autonomous, assigning liability becomes complex. Understanding whether the dentist, AI developer, or both should be held responsible requires nuanced legal discussions and potentially new legislative frameworks. Determining who is responsible, the dentist, the AI developer, or both, remains a gray area, necessitating clear legislation and guidelines.

Algorithmic Fairness and Bias

Algorithmic fairness is another significant ethical challenge. AI models learn from the data they are trained on. AI models can make unfair or discriminatory diagnoses if the data are biased. Ensuring AI systems are trained on diverse and representative data is critical for mitigating this issue. Ensuring diversity and representativeness in training data is an essential ethical consideration [39]. The application of AI in dental caries diagnosis holds immense promise, but it is not without ethical and legal challenges [40]. As we continue to integrate AI into dental practice, an ethical and legal framework tailored to the unique challenges of AI is essential. This effort will be instrumental in building a future where AI is integral to dentistry, driving better patient outcomes while maintaining trust and upholding ethical principles.

Existing artificial intelligence technologies for dental caries diagnosis

Several AI technologies are currently being employed or developed for dental caries diagnosis.

Pearl®

Pearl, an innovative AI-powered dental radiograph interpretation tool, is revolutionizing the field of dental diagnostics. Pearl mitigates these risks by bringing precision and consistency to enhance diagnostic accuracy and standardization across dental practices [41]. At the heart of the functionality of Pearl is its sophisticated deep learning model trained on a vast array of dental radiographs. The tool can identify and annotate several dental pathologies, including but not limited to dental caries, periodontal bone loss, and periapical lesions. By recognizing these common dental conditions, Pearl acts as an invaluable second set of eyes for the dentist, ensuring that no minor detail is overlooked [42]. While Pearl can aid in diagnosis, it is not a replacement for the clinical judgment of a dentist. The tool should be seen as an aid to support dental professionals rather than a standalone diagnostic system [43].

Overjet

Overjet is a pioneering tool in dental diagnostics that uses advanced AI technology to interpret dental imaging. This remarkable tool transforms dental care by offering greater accuracy, consistency, and efficiency in the analysis and interpretation of dental radiographs. In addition, the AI-driven technology of Overjet significantly reduces this variability by bringing precision and standardization that can greatly enhance diagnostic accuracy and consistency across dental practices. The cornerstone of Overjet’s effectiveness lies in its innovative deep learning algorithms trained on an extensive collection of dental radiographs [44]. These algorithms allow Overjet to identify and annotate dental conditions, including dental caries, periodontal disease, and other common dental pathologies. As such, Overjet can serve as a highly reliable second opinion for the dentist, ensuring no detail is overlooked [45].

Denti. AI®

Denti. AI is an advanced AI tool explicitly designed to transform how dental professionals diagnose and manage dental caries, which is one of the most common oral health problems globally. By harnessing cutting-edge AI algorithms and machine learning models, Denti. AI is making dental caries detection and treatment planning more accurate and efficient. Traditionally, diagnosing dental caries has primarily depended on a dentist’s expertise, experience, and quality of dental radiographs [46]. While generally effective, this process can be somewhat subjective and subject to variability and human error. Denti. AI technology addresses these limitations by providing a more objective, standardized analysis of dental radiographs [15].

Future perspectives on artificial intelligence-based caries detection and diagnosis

As we look toward the future of dentistry, the role of AI in dental caries detection and diagnosis appears promising. The ability of AI to process large volumes of data quickly and accurately has already begun to transform caries diagnosis, making it more efficient and precise. We might see AI systems becoming increasingly sophisticated in the future, learning from each new case they encounter and continuously improving their diagnostic capabilities. The integration of AI-based diagnosis into everyday dental practice will also advance. More dental clinics, hospitals, and schools will likely adopt these technologies. As AI systems become more accessible and user-friendly, it will be easier for dental professionals to incorporate them into their practices. Continued research and innovation in AI technology and interdisciplinary collaboration among AI experts, dental professionals, ethicists, and legal experts will be essential for navigating these challenges.

## Conclusions

The future of AI-based caries detection and diagnosis is exciting. AI can revolutionize dentistry with its potential to enhance accuracy, improve efficiency, and personalize care. However, this future also requires careful navigation of ethical, legal, and technical challenges. By tackling these issues head-on, we can harness the power of AI to improve oral health and reshape the future of dental care.
